# Supplementary material for: The causal role of circulating inflammatory markers in osteoporosis: a bidirectional Mendelian randomized study
Source: Front Immunol. 2024 Jul 18;15:1412298. doi: 10.3389/fimmu.2024.1412298 (PMC11291241; doi:10.3389/fimmu.2024.1412298)
Supplement: Supplementary file 1 [file DataSheet_1.zip › supplementary material/CIM-OSTEOPOROSIS /GCST90274795.csv_leave_one_out.pdf]

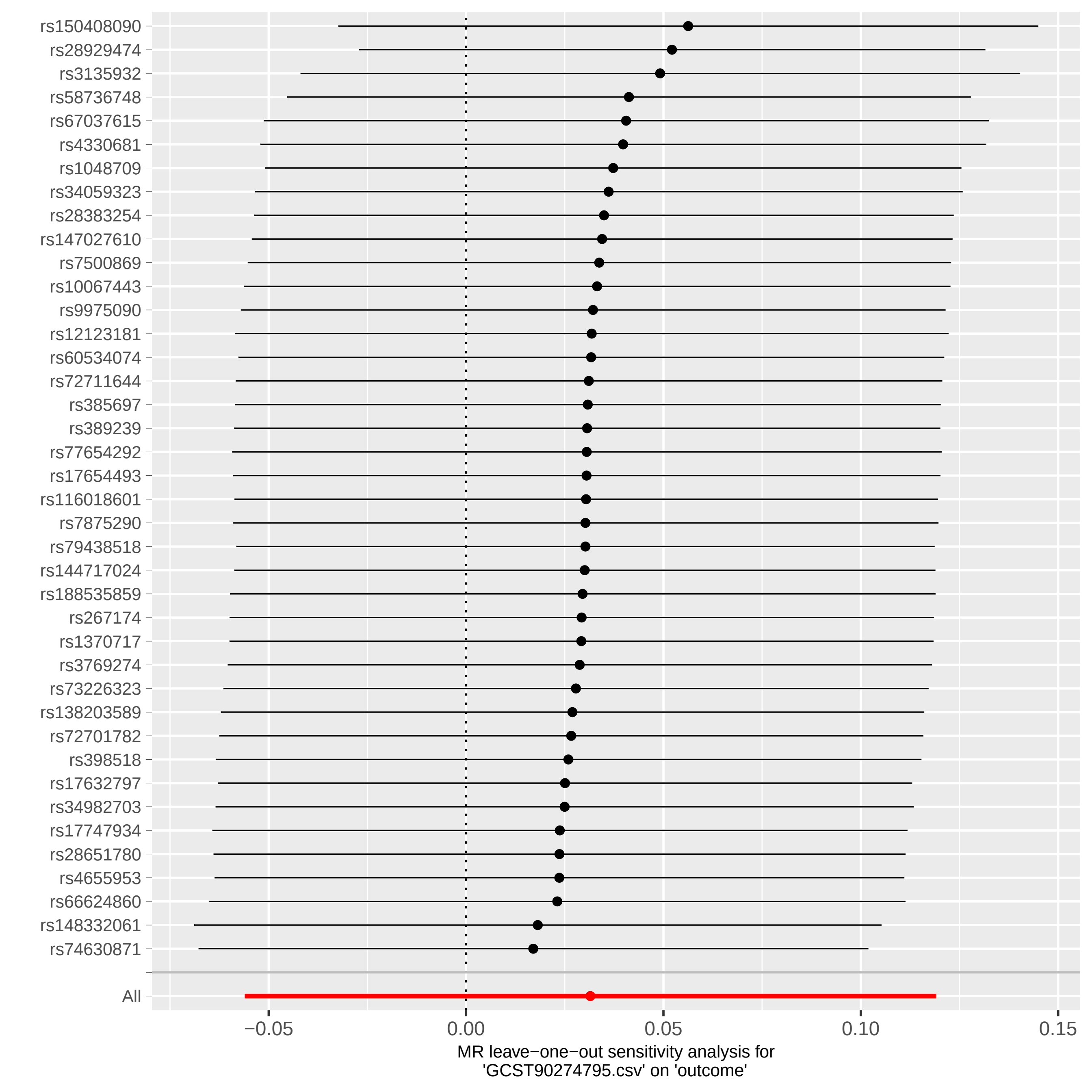
rs150408090
rs28929474
rs3135932
rs58736748
rs67037615
rs4330681
rs1048709
rs34059323
rs28383254
rs147027610
rs7500869
rs10067443
rs9975090
rs12123181
rs60534074
rs72711644
rs385697
rs389239
rs77654292
rs17654493
rs116018601
rs7875290
rs79438518
rs144717024
rs188535859
rs267174
rs1370717
rs3769274
rs73226323
rs138203589
rs72701782
rs398518
rs17632797
rs34982703
rs17747934
rs28651780
rs4655953
rs66624860
rs148332061
rs74630871
All
−0.05
0.00
0.05
0.10
0.15
MR leave−one−out sensitivity analysis for
'GCST90274795.csv' on 'outcome'
